# The Impact of COVID-19 Home Confinement on Mexican University Students: Emotions, Coping Strategies, and Self-Regulated Learning

**DOI:** 10.3389/fpsyg.2021.642823

**Published:** 2021-04-28

**Authors:** Martha Leticia Gaeta, Laura Gaeta, María del Socorro Rodriguez

**Affiliations:** Facultad de Educación, Universidad Popular Autónoma del Estado de Puebla, Puebla, Mexico

**Keywords:** COVID-19, coping strategies, self-regulated learning, online learning, higher education

## Abstract

One of the main challenges in higher education is promoting students' autonomous and self-regulated learning, which involves managing their own emotions and learning processes in different contexts and circumstances. Considering that online learning during the COVID-19 pandemic may be an opportunity for university students to take greater responsibility for their learning, it is essential to explore the strategies they have developed in the face of emotional and learning challenges during the health crisis. This study aimed at analyzing the relationships between students' emotions, coping strategies, and self-regulated learning in online learning during COVID-19 home confinement. The participants were 1,290 Mexican students from different universities throughout the country, who answered an online self-report questionnaire from standardized instruments adapted to the pandemic. Data were analyzed with descriptive and inferential analyses, including a structural equation model (SEM). Findings indicate that, although anxiety, boredom, and frustration were present among participants during confinement, the primary emotions were gratitude, joy, and hope. Second, the main coping strategies used by students participating were focused on facing and reassessing the situation. Furthermore, tranquility, hope, gratitude, and joy were positively related to self-regulated learning, although, loneliness and disinterest were negatively related. Finally, it was found that an approach to coping strategies mediated the relationship between emotions and self-regulated learning. Thus, teachers should help students understand the relevance of active coping strategies and use student-centered learning models that promote autonomous and self-regulated learning, considering each learner's needs, during and after confinement.

## Introduction

The health crisis due to the new coronavirus SARS-CoV-2 has affected the health and lives of millions of people worldwide in just a few months (WHO, 2020). In light of this scenario, preventive health actions in different countries have been crucial. However, the impact on people's lives, including education, has also been critical. In Mexico, the confinement measures necessary to fight the spread of COVID-19 have caused closing educational institutions nationwide since the end of March 2020. Therefore, in most universities, digital technology has been seen as a means to “solve” space-time limitations (Ordorika, 2020).

The abrupt change to online learning has emphasized the need for students to self-regulate their learning; to be responsible for their studies and have constant habits to persevere in their academic goals (Martínez-Sarmiento and Gaeta, 2019). The need to adopt a more active role in the learning process, and to permanently update this throughout one's life, has been emphasized for some time (Delors, 1996) and is reflected in the “Latin American new educational agenda: the 2030 objectives” (Martín and Jabonero, 2017). This condition becomes even more necessary when we know the crisis caused by COVID-19 will reconfigure all structures established until now, although we do not know to what degree.

University students must therefore develop second-order learning capabilities and activate their cognitive, motivational, and effective learning strategies to self-regulate their learning (Zimmerman, 2008), to face academic demands and responsibilities in light of their future professional activities. However, during the home confinement that has been required during the COVID-19 pandemic, not all young people have had the same living conditions, adequate access to technology, nor do they have similar behavior and skills to face these conditions. Moreover, the content of some disciplinary areas is not adaptable to virtual settings (Pinzón and Hederich, 2008). Besides, each student has a particular way of facing social isolation due to home confinement; thus, in some cases, students' emotional well-being may be significantly reduced upon not knowing how to face uncertainty, anxiety, boredom, and even the sadness that staying home implies.

This complex reality has led researchers to analyze the educational experiences of university students during the COVID-19 lockdown. However, as far as we know, there is still not much evidence regarding students' emotions and coping strategies in terms of learning and self-regulation processes in online learning.

### Emotions in University Students During Home Confinement

Emotional experiences involve a wide range of physiological, functional, and social/affective aspects (Paoloni, 2014) to respond to the demands of different contexts. Life events that affect everyday experiences have a strong emotional impact (Sandín and Chorot, 2003); they may be considered positive or negative, depending on the person's evaluation of lived experience (Bisquerra, 2011). However, in situations seen as highly threatening due to their uncertain or uncontrollable nature, people tend to present a combination of several emotional reactions (Lazarus and Folkman, 1984).

According to literature, positive emotions need to be encouraged, since they expand the intellectual, physical, and social resources of individuals, increasing the reserves from which they can draw when threats or opportunities arise. Moreover, these emotions activate avoidance and approach behaviors and contribute to more creative and open thinking and behavior, which allows building resources that serve in future adverse situations, that is, coping strategies (Fredrickson, 2001), which are very important for people's well-being (Lazarus and Folkman, 1984).

According to Taylor (2019), there tend to be different reactions in all pandemics, such as fear, anxiety, stress, confusion, and anger. Likewise, those who spend time in isolation tend to show symptoms related to post-traumatic stress, for example, fear and anxiety (Brooks et al., 2020). As indicated by several researchers (Johnson et al., 2020; González-Jaimes et al., 2021), the COVID-19 lockdowns have had a significant emotional impact on young people, causing issues such as sleeping problems, high levels of stress, anxiety, fear, anguish, and uncertainty.

Sandin et al. (2020) found that the COVID-19 health crisis had affected young Spanish people aged 19–30 years, manifesting as emotions such as concern, nervousness, unease, despair, or depression, mainly derived from intolerance to uncertainty. However, home confinement has also favored positive personal experiences, such as a greater interest in people and their future. These results coincide in general with those from Johnson et al. (2020), observing the pandemic has caused different emotional states among Argentinean adults; uncertainty, fear, and distress but also feelings of responsibility, care, and value for others.

Regarding academic life, during the COVID-19 pandemic young people have experienced uncertainty, stress, and concern for their studies since they had to carry them online, which has affected their emotional well-being (Orellana and Orellana, 2020) and academic performance (Lozano-Díaz et al., 2020). Cao et al. (2020) conducted research with 7,143 Chinese university students and concluded that 21.3% showed mild symptoms of anxiety. Moreover, living with parents in urban zones and having economic stability contributed to mitigating anxiety in young people, while the delay in academic activities contributed to an increase in anxiety during the lockdown.

### Stress Coping Strategies

From the conception of stress as a process, people faced with the same stressful situation use different strategies that may result in them being adaptive or non-adaptive according to how they interpret the situation. These include being influenced by personal traits, perceptions of themselves, previous experiences, and depend on the type of stressor (Lazarus and Folkman, 1986). Thus, coping refers to the behavioral and cognitive effort used by a person to face situations that are considered stressful (Lazarus and Folkman, 1984; Sandín and Chorot, 2003), with there being a strong bond between coping strategies and the specific situation (García-Arroyo et al., 2019).

Studies and exploration of this subject traditionally differentiate between the cognitive coping efforts undertaken. From this perspective, some strategies are thought to be focused on the problem (person-situation relationship) and others focused on the emotion or emotional alterations derived from the stressful situation (Lazarus and Folkman, 1984, 1986; Amin et al., 2019). Strategies focused on the problem may be divided into active coping, which seeks to modify the situation or lessen its effects, and delayed coping, which involves looking for the appropriate opportunity to act (Lazarus and Folkman, 1986). On the other hand, emotional strategies may take a different course and some of them serve only momentarily since, if maintained for an extended period, they may prevent adequate adaptation or the realization of active efforts, for example, when anger is released openly or when there is a denial of the situation. Emotion-focused strategies can also be differentiated into active and passive strategies.

More recent research has made a distinction between cognitive and behavioral coping strategies (Billings and Moos, 1981). The former are aimed at mediating between the stressors and the consequences experienced, while the latter involves specific actions to manage the situation or the emotional response. From this perspective, the approach (active) or avoidance (passive) aspects of the problematic situation have also been considered (Billings and Moos, 1981). Active cognitive coping would focus on evaluating the situation and previous strategies used, while active behavioral coping would focus on dealing directly with the stressful situation and its effects. In turn, avoidance of cognitive coping would imply putting the problem aside. On the other hand, emotion-focused coping, regardless of focusing on cognitive or behavioral strategies, would be aimed at re-establishing emotional balance in some way.

It should be noted that this perspective is compatible with the former classification (problem/emotion) (Lazarus and Folkman, 1984) and is reflected in specific measurement instruments that consider these two dimensions (Moos, 1993): approach coping and avoidance coping; the cognitive type strategies (such as positive reevaluation or problem solving), or behavioral type strategies (such as seeking social support) are considered. It is important to mention that these dimensions are not mutually exclusive. Moreover, coping strategies are not good or bad in themselves; their effectiveness depends on the uncertainty or intensity of the stressful situation, among other factors (Lazarus and Folkman, 1986). However, some strategies, such as problem-centered strategies, tend to be most desirable (Folkman and Lazarus, 1980), while avoidance strategies are the least desirable (Lazarus, 2006). As Vizoso (2019) mentions, problem centered coping strategies have shown a positive relationship with resilience; hence, when faced with the current pandemic crisis, students must hold on and actively flourish.

Coping strategies have to be considered within a specific context, depending on the situation to which the person is exposed (Lazarus, 2006). The COVID-19 pandemic presents itself accompanied by stressors such as the illness itself, lockdown, possible economic difficulties, and different academic or labor scenarios. In light of this complicated situation, it is relevant to analyze university students' coping strategies for their emotional stability and academic performance.

### Self-regulation of Learning in Online Environments

Self-regulated learning is one of the most efficient types of learning in varied educational contexts and for different levels. It is an active process through which students establish their learning objectives and try to monitor and manage their feelings and behavior through strategies and actions that are cyclically planned and adapted for the achievement of their learning objectives (Zimmerman, 2008). Therefore, it constitutes an essential factor for involvement and academic success in on-site (Torrano et al., 2017) and online environments (Cerezo et al., 2015; Berridi and Martínez, 2017).

The online learning environments, as indicated in the systematic review of Wong et al. (2019), imply a highly autonomous component and one of self-management of the students to make their own decisions about what materials to review, when or how much to study, and what strategies to modify for the achievement of their academic goals. Likewise, more significant effort is necessary to maintain attention and focus during online activities (Alghamdi et al., 2020). Therefore, self-regulation of learning is considered a fundamental process that allows students to adapt to contexts, in this case to online learning, allowing them to develop planning, prevision, and monitoring activities (Barnard-Brak et al., 2010; Cerezo et al., 2015), to persevere when faced with difficulties in the achievement of their academic goals.

Self-regulated learning involves metacognitive processes, learning skills, and the management of emotional reactions. It thus constitutes an essential process for psychological functioning (Schmeichel and Baumeister, 2004; Zimmerman, 2008). It is the complex and dynamic interaction between the affective-motivational and the cognitive aspects that influence the effort and performance of the academic task (Zimmerman, 2008; Hendrie and Bastacini, 2020). This research corresponds to the achievement of learning in online environments and in lockdown conditions, which involves high-stress levels of coping.

According to Boekaerts (2002), a stressful factor may unleash very intense emotions in some young people and moderate emotions in others; hence, having effective control mechanisms helps students to protect themselves against the emotional states that arise as a result of daunting circumstances (Koole and Jostmann, 2004). Several researchers (De la Fuente et al., 2015) have found a significant relationship between self-regulated learning and the academic stress coping strategies of university students. They found a stronger relationship with coping strategies focused on the problem. On this subject, Jackson et al. (2000) state that optimal coping skills and regulating behavior, particularly in situations of stress, should be analyzed within an interpersonal context.

### Purpose of the Study

Together with the crisis caused by the COVID-19 pandemic, the formative process of attending university requires students to put into practice functional strategies that enable young people to maintain their emotional well-being and desirable performance (Lozano-Díaz et al., 2020). According to literature, the positive and negative emotions experienced during stressful situations reflect a person's momentary evaluation of their well-being (Lazarus and Folkman, 1984). Correspondingly, various researchers have documented the significant relationship between different coping strategies and emotional well-being (Vizoso, 2019), and between the former and academic performance (Gargantiel, 2018). Moreover, the relationship between coping strategies and self-regulated learning in a stressful academic context has been found (De la Fuente et al., 2015), highlighting the relevance of analyzing the antecedents and consequences of coping strategies. Thus, in line with previous research, this study aimed to analyze the relationships between students' emotions, coping strategies, and self-regulated learning in an online learning environment during COVID-19 home confinement. It specifically sought to:

- Identify the students' living conditions during home confinement
- Acknowledge the emotions experienced by students
- Identify students' coping strategies
- Examine the relationships between students' emotions, coping strategies, and self-regulated learning
- Examine the possible mediating role of coping strategies between emotions and self-regulated learning.

## Materials and Methods

### Participants

Participants were 1,290 undergraduate (84.2%) and graduate (15.8%) students from different Mexican universities, with an average age of 24.22 years (*SD* = 7.98). From the total sample, 906 were females (70.2%). While 828 (64.2%) students were enrolled in private institutions, 462 (35.8%) were enrolled in public institutions. Regarding the discipline area, 24.3% were studying Social Sciences, Administration and Law programs; 20.2% in Education; 18.6% in Health Sciences; 8.1% in Natural and Exact Sciences; 6.4% in Engineering and Manufacture; 3.7% in Arts and Humanities; 1.5% in Agronomy and Veterinary Sciences; and the rest of participants (17.2%) were studying in other programs.

### Variables and Instruments

A self-report questionnaire was designed to measure students' emotions, coping strategies, and self-regulated learning. Additionally, *ad-hoc* questions on the students' background variables and living conditions during the COVID-19 lockdown were included.

Regarding emotions, the students selected from a list of 15 emotions-compassion, tranquility, loneliness, anger, frustration, hope, boredom, fear, gratitude, confusion, sadness, anxiety/concern, lack of interest, joy, despair- (Pekrun et al., 2005; Bisquerra, 2009) in what extent they had experienced any of them during the lockdown. Answers were rated on a 5-point Likert scale (1 = never to 5 = always).

Emotional coping strategies were assessed through two instruments, the first was the Mexican version (González and Landero, 2007) of *Stress Coping Questionnaire* (Sandín and Chorot, 2003). This scale is divided amongst seven dimensions: (a) Focalization on Problem-Solving (FPS; e.g., *I have dealt with the problem by proposing several concrete solutions*; α = 0.71); (b) Negative Self-focalization (NSF; e.g., *I have resigned myself to accepting things as they are*; α = 0.55); (c) Positive Reappraisal (PRE; e.g., *In spite of everything, I have verified that things could have turned out worse*; α = 0.66); (d) Open Emotional Expression (OEE; e.g., *I have thanked some people*; α = 0.71); (e) Avoidance (AVD; e.g., *I've tried to forget everything*; α = 0.50); (f) Seeking Social Support (SSS; e.g., *I have spoken with friends or family to reassure myself when I have felt unwell*; α = 0.75); (g) turning to Religion (RLG; e.g., *I have performed some ritual such as lighting candles and praying;* α = 0.66). The adapted version for this study is formed by 21 items (from the 42 original items), three items per dimension, with Cronbach's alpha value (α) = 0.80. Answers were rated on a 5-point Likert scale.

The second instrument for measuring emotional coping strategies used the Argentinean version (Ongarato et al., 2009) of the *Coping Resources Inventory* (Moos, 1993). The scale is divided amongst four dimensions: (a) Cognitive Coping Approach (CCAP; four items; e.g., *I have thought about how this situation can change my life for the better*; α = 0.65); (b) Behavioral Coping Approach (BCAP; 3 items; e.g., *I have sought help from other people or groups*; α = 0.69); (c) Cognitive Coping Avoidance (CCAV; three items; e.g., *I've lost hope that things will go back to the way they were;* α = 0.55); (d) Behavioral Coping Avoidance (BCAV; three items; e.g., *I started reading to entertain myself*; α = 0.50). The adapted version for this study is formed by 13 items (from the 22 original items), with Cronbach's alpha value (α) = 0.80. Answers were rated on a 5-point Likert scale.

Self-regulated Learning was assessed using the *Self-Regulation Formative Questionnaire* (Gaumer Erickson and Noonan, 2018). The scale, compounded by 22 Likert-Type items, measures the students' perceived competence in the four essential components of self-regulation: (a) planning and articulation of the objective to achieve (five items; e.g., *I plan the projects I want to carry out*; α = 0.76); (b) Monitoring of the progress and the interferences regarding the objective (six items; e.g., *I know when I'm behind on a project*; α = 0.77); (c) control of the changes to make when things do not go as planned, through the implementation of specific strategies (six items; e.g., *I try all the possibilities that are necessary to be successful*; α = 0.76); (d) reflect on the results and opportunities for improvement (five items; e.g*., I think about how well I am doing my work*; α = 0.75). The total scale Cronbach's alpha value (α) = 0.92.

The students' contextual and academic variables were collected through multiple choice questions. They included gender, age, university type (public/private), education level (undergraduate/postgraduate), and academic discipline. Regarding students' living conditions, questions included state of the country (coded by region) and the environment (urban, semi-urban, and rural) in which students lived during the lockdown, the people with whom they shared living spaces (family, friends/acquaintances, lived alone), the housing spaces and technological resources to continue with their studies online.

### Procedure

The questionnaire was distributed to the students through the Google Forms tool. In some cases, it was distributed through institutional mail and in others through different social, academic, and research group networks. Participants were fully informed about the study's objectives and gave their consent to voluntarily and anonymously answer the instrument, in accordance with the Declaration of Helsinki. This study was approved by the University Ethics Committee (March 1, 2020). The information was collected during June and July 2020.

### Data Analysis

The data were analyzed to verify that there was no missing or atypical information and to examine the normality and linearity of the measures. From an initial sample of 1,327 students, 37 were eliminated since they had many missing data or presented atypical values. The final sample contained 1,290 students. First, descriptive analyses (frequencies, central tendency, and dispersion) and non-parametric bivariate correlations (given the data non-normality) were carried out through SPSS Statistics. Second, a structural equation model was analyzed (Hair et al., 2010), using the SmartPLS 3.0 statistical program.

## Results

### Students' Living Conditions During Home Confinement

Regarding living conditions during the COVID-19 lockdown, most participants indicated living in states of the country's central region (57.1%; *n* = 736). Table 1 presents the distribution of the students by country region.

**Table 1:** Students' distribution by country region.

**Country region**	***n***	**%**
Center	736	57.1
South-southeast	314	24.3
Northwest	170	13.2
West	57	4.4
Northeast	13	1
Total	1,290	100

The majority of the students' housing (69.0%; *n* = 890) was located in an urban area, and only 9.8% lived in a rural area (*n* = 127). The majority of the students reported living with family members (95.5%; *n* = 1,232) and only 2.6% (*n* = 33) reported living alone. The majority of the students indicated having a personal space for studying (78.3%; *n* = 1,010) and light and natural ventilation (66.0%; *n* = 852). A high percentage of students (92.9%; *n* = 1,119) had Internet and a computer or other electronic devices to continue with their studies online (92.9%; *n* = 1,119).

### Students' Emotions

The emotions reported with greater frequency by participants were gratitude (*M* = 3.72; *SD* = 1.08), joy (*M* = 3.50; *SD* = 0.95), and hope (*M* = 3.39; *SD* = 1.08), followed by other emotions such as anxiety (*M* = 3.33; *SD* = 1.17), boredom (*M* = 3.24; *SD* = 1.21), and frustration (*M* = 3.21; *SD* = 1.05). The emotions least reported were fear (*M* = 2.71; *SD* = 1.19), loneliness (*M* = 2.63; *SD* = 1.18), and lack of interest (*M* = 2.51; *SD* = 1.19).

### Coping Strategies Used by Students

Most participants (74%) mentioned using Positive Reappraisal of the Situation (PRE) and 67% indicated having put Cognitive Coping Approach (CCAP) strategies into practice. Also, 67% of participants carried out Avoidance (AVD) strategies, followed by 64% that mentioned having used Focalized on Problem-Solving (FPS) strategies, and 63% that used Behavioral Coping Avoidance (BCAV) Strategies.

### Relationships Between Emotions, Coping Strategies, and Self-Regulated Learning

Upon considering the coping strategies based on approach or avoidance (see Table 2), it was found that Cognitive Coping Approach (CCAP) strategies correlate positively with emotions such as compassion, hope, gratitude, and joy. However, they also correlate positively with fear and confusion. Cognitive Coping Avoidance (CCAV) strategies correlate positively with all negative emotions (loneliness, anger, frustration, boredom, fear, confusion, sadness, anxiety/concern, lack of interest, and despair), and negatively with tranquility. Behavioral Coping Approach (BCAP) strategies correlate positively with emotions such as confusion, fear, and despair, and to a lesser extent, with loneliness, compassion, and frustration. Behavior Coping Avoidance (BCAV) and Avoidance (AVD) strategies correlate with positive and negative emotions.

**Table 2:** Bivariate correlations between emotions, coping strategies, and self-regulated learning (*n* = 1,290).

**/Coping strategies**	**CCAP**	**BCAP**	**CCAV**	**BCAV**	**FPS**	**NSF**	**PRE**	**OEE**	**AVD**	**SSS**	**RLG**
**Positive emotions:**											
Compassion	0.23^**^	0.16^**^	0.07*	0.12^**^	0.21^**^	0.04	0.23^**^	0.03	0.10^**^	0.21^**^	0.16^**^
Tranquility	0.07*	−0.05	−0.23^**^	0.05	0.25^**^	−0.23^**^	0.25^**^	−0.26^**^	0.07*	−0.06*	0.05
Hope	0.29^**^	0.13^**^	−0.07^**^	0.20^**^	0.32^**^	−0.13^**^	0.39^**^	−0.09^**^	0.14^**^	0.13^**^	0.30^**^
Gratitude	0.35^**^	0.13^**^	−0.03	0.15^**^	0.27^**^	−0.05	0.42^**^	−0.02	0.18^**^	0.16^**^	0.20^**^
Joy	0.20^**^	0.10^**^	−0.08^**^	0.14^**^	0.24^**^	−0.16^**^	0.33^**^	−0.10^**^	0.15^**^	0.16^**^	0.20^**^
**Negative emotions:**											
Boredom	0.00	0.08^**^	0.24^**^	0.08^**^	−0.19^**^	0.29^**^	−0.13^**^	0.29^**^	0.15^**^	0.07*	−0.07^**^
Loneliness	0.04	0.17^**^	0.34^**^	0.11^**^	−0.06*	0.38^**^	−0.08^**^	0.29^**^	0.12^**^	0.12^**^	−0.05
Anger	0.08^**^	0.12^**^	0.29^**^	0.04	−0.09^**^	0.33^**^	−0.07*	0.52^**^	0.10^**^	0.14^**^	0.00
Frustration	0.15^**^	0.14^**^	0.39^**^	0.12^**^	−0.07*	0.44^**^	−0.02	0.45^**^	0.12^**^	0.19^**^	−0.01
Fear	0.20^**^	0.22^**^	0.38^**^	0.16^**^	−0.02	0.39^**^	0.00	0.31^**^	0.14^**^	0.22^**^	0.14^**^
Confusion	0.19^**^	0.24^**^	0.41^**^	0.16^**^	0.02	0.40^**^	0.01	0.36^**^	0.15^**^	0.22^**^	0.05
Sadness	0.12^**^	0.19^**^	0.41^**^	0.14^**^	−0.09^**^	0.44^**^	−0.05	0.40^**^	0.10^**^	0.21^**^	0.00
Anxiety/Concern	0.18^**^	0.19^**^	0.40^**^	0.16^**^	−0.02	0.43^**^	0.01	0.42^**^	0.15^**^	0.20^**^	0.00
Disinterest	0.05	0.15^**^	0.28^**^	0.07*	−0.09^**^	0.33^**^	−0.12^**^	0.33^**^	0.09^**^	0.10^**^	−0.02
Despair	0.13^**^	0.21^**^	0.34^**^	0.11^**^	−0.09^**^	0.44^**^	−0.06*	0.45^**^	0.13^**^	0.20^**^	0.04
**Self-regulated learning:**											
Planning	0.21^**^	0.04	−0.13^**^	0.208^**^	0.41^**^	−0.18^**^	0.34^**^	−0.18^**^	0.13^**^	0.08^**^	0.17^**^
Monitoring	0.26^**^	0.04	−0.09^**^	0.18^**^	0.34^**^	−0.11^**^	0.37^**^	−0.10^**^	0.17^**^	0.08^**^	0.14^**^
Control	0.24^**^	0.01	−0.15^**^	0.18^**^	0.33^**^	−0.21^**^	0.37^**^	−0.19^**^	0.15^**^	0.07*	0.15^**^
Reflection	0.31^**^	0.06*	−0.08^**^	0.24^**^	0.35^**^	−0.13^**^	0.43^**^	−0.12^**^	0.20^**^	0.14^**^	0.20^**^

*CCAP, Cognitive Coping Approach; BCAP, Behavioral Coping Approach; CCAV, Cognitive Coping Avoidance; BCAV, Behavior Coping Avoidance; FPS, Focalization in Problem-Solving; NSF, Negative Self-focalization; PRE, Positive Reappraisal; OEE, Open Emotional Expression; AVD, Avoidance; SSS, Seeking Social Support; RLG, Turning to Religion;*

** *p < 0.01*.

Cognitive Coping Approach (CCAP) strategies, Cognitive Coping Avoidance (CCAV) strategies, and Behavior Coping Avoidance (BCAV) strategies correlate positively with all the Self-regulated Learning dimensions, the first and third and the second negatively. Behavioral Coping Approach (BCAP) strategies only correlate significantly with reflection, with very little strength.

A positive correlation was found between Focalization in Problem-Solving (FPS) and Positive Reappraisal (PRE) with all the positive emotions (compassion, tranquility, hope, gratitude, and joy). On the other hand, a positive correlation was found between Negative Self-Focalization (NSF) and Open Emotional Expression (OEE) with all the negative emotions (boredom, loneliness, anger, frustration, fear, confusion, sadness, anxiety/concern, and despair). Seeking Social Support (SSS) correlated positively with emotions associated with uncertainty, such as fear and confusion, and compassion, sadness, despair, and to a lesser extent with gratitude and joy. Finally, Turning to Religion (RLG) correlated positively with hope, joy, gratitude, compassion, and fear.

Focalization on Problem-Solving (FPS) and Positive Reappraisal (PRE) present a strong correlation with all the Self-regulated Learning dimensions. Seeking Social Support (SSS) also correlates with all the Self-regulated Learning dimensions, although the relationship's strength is meager. Negative Self-focalization (NSF) and Open Emotional Expression (OEE) show a negative correlation with the Self-regulated Learning dimensions. This inverse relationship also happens in the case of Cognitive Avoidance strategies. Finally, Turning to Religion (RLG) correlates with the Self-regulated Learning dimensions, although the relationship's strength is also low.

### Mediation Analysis of Coping Strategies

#### Hypothetical Model

**Figure 1:**
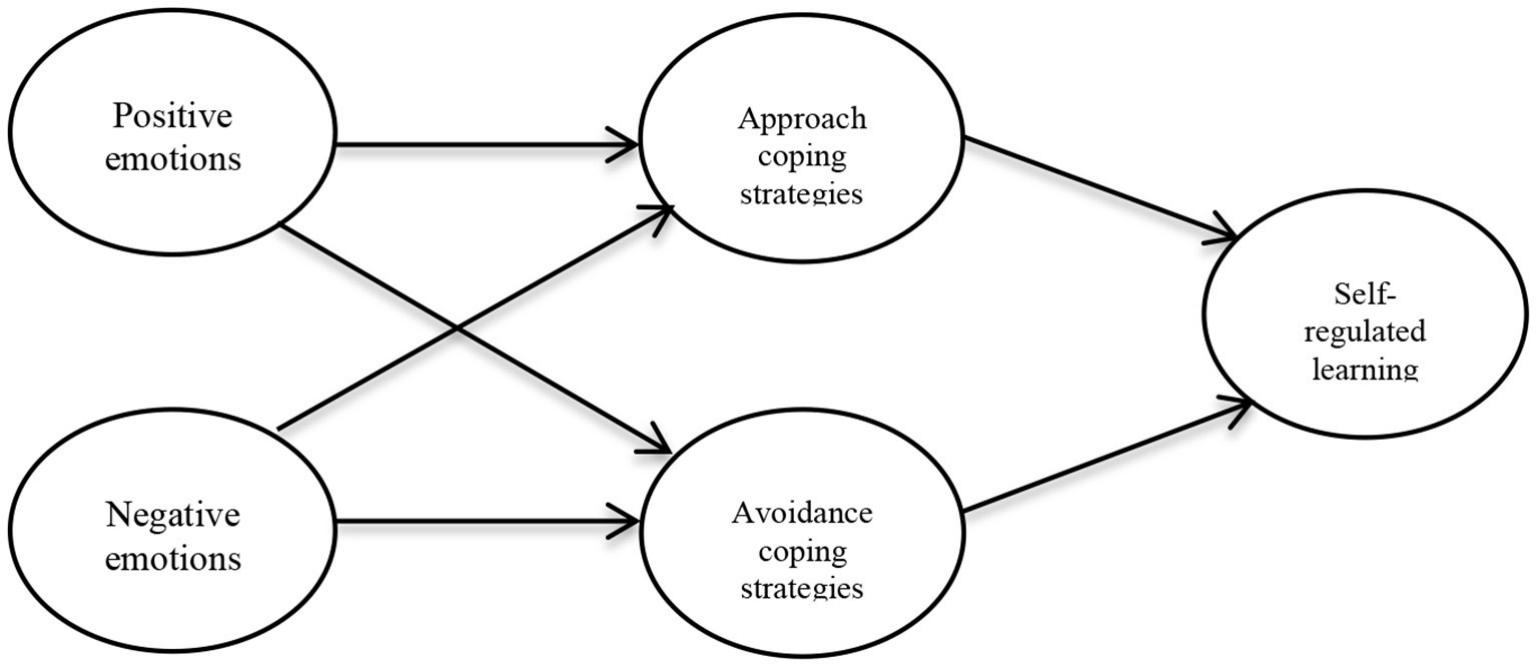
Hypothetical mediation model of coping strategies between emotions and self-regulation of learning.

The correlation analysis showed that the majority of sub-scales correlated with each other, and allowed to verify the mediation assumptions in the structural equation model. A mediation analysis was carried out considering emotions as a predictor variable, self-regulated learning as a dependent variable, and coping strategies as a mediator (see Figure 1).

In line with the literature (Billings and Moos, 1981; Lazarus and Folkman, 1984, 1986; Moos, 1993; De la Fuente et al., 2015), the following hypotheses were set for the model:

H1: *Students' positive emotions* are positively correlated with approach coping strategies and negatively with avoidance coping strategies.
H2: *Students' negative emotions* are positively correlated with avoidance coping strategies and negatively with the approach coping strategies.
H3: *Students' self-regulated learning* is positively correlated with approach coping strategies and negatively with avoidance coping strategies.
H4: *Students' coping strategies* mediate the relationship between emotions and self-regulated learning.

#### Evaluation of the Mediation Model

As shown in Table 3, Cronbach's alpha (α) indicates good internal consistency of the model, with values between 0.70 and 0.90 (Celina and Campo-Arias, 2005). Regarding the Average Extracted Variance (AVE) and the Compounded Reliability (CR), results show AVE values that exceed the established threshold of 0.50 (Fornell and Larcker, 1981) and CR values that exceed the established threshold of 0.70 (Nunnally, 1978).

**Table 3:** Measurement model results.

**Dimension**	**Cronbach's** **alpha(α)**	**AVE**	**Compounded** **Reliability (CR)**
Positive emotions	0.69	0.62	0.83
Negative emotions	0.89	0.64	0.92
Approach coping strategies	0.73	0.55	0.83
Avoidance coping strategies	0.70	0.63	0.83
Self-regulated learning	0.92	0.54	0.93

To analyze the discriminant validity, the Heterotrait-Monotrait Ratio (HTMT) was used; values must be below the threshold of 0.90 (Henseler et al., 2015). Results show that all the constructs met the HTMT criterion (Table 4).

**Table 4:** Heterotrait-Monotrait Ratio (HTMT) results.

	**1**	**2**	**3**	**4**	**5**
1. Positive emotions	–				
2. Negative emotions	0.17	–			
3. Approach coping strategies	0.72	0.14	–		
4. Avoidance coping strategies	0.18	0.83	0.17	–	
5. Self-regulated learning	0.45	0.07	0.59	0.11	–

Bootstrapping technique with 500 samples was used to determine the significance of the path coefficients (β). Table 5 presents the measurement model results. There were significant direct effects from positive emotions on approach coping strategies (β = 0.52, *p* = 0.000), and of the latter on self-regulated learning (β = 0.49, *p* = 0.000). The indirect effect of the model was also significant (β = 0.25, *p* = 0.000). These results show that when approach coping strategies are considered as a mediator variable, reduction of the direct effect of positive emotions on approach coping strategies, and the direct effect of the latter on self-regulated learning occurs. Therefore, approach coping strategies significantly partially mediate between emotions and students' self-regulated learning. Findings also indicate that the mediation model explained 25% of the variability in students' self-regulated learning.

**Table 5:** Measurement model results.

**Effect**	**β**	***t***	***SE***
**Direct effect**			
Positive emotions → Approach coping strategies	0.52	19.87^***^	0.03
Positive emotions → Avoidance coping strategies	−0.05	2.26^*^	0.02
Negative emotions → Approach coping strategies	−0.02	0.76	0.03
Negative emotions → Avoidance coping strategies	0.65	37.40^***^	0.02
Approach coping strategies → Self-regulated learning	0.49	22.03^***^	0.02
Avoidance coping strategies → Self-regulated learning	−0.04	1.67	0.03
**Indirect effect**			
Positive emotions → Approach coping strategies → Self-regulated learning	0.25	14.13^***^	0.02
Positive emotions → Avoidance coping strategies → Self-regulated learning	0.00	1.26	0.00
Negative emotions → Approach coping strategies → Self-regulated learning	−0.01	0.76	0.01
Negative emotions → Avoidance coping strategies → Self-regulated learning	−0.03	1.66	0.02
Self-regulated learning Total effect model (*R*^2^ = 0.25)			

* *p < 0.05*,

*** *p < 0.001*.

**Figure 2:**
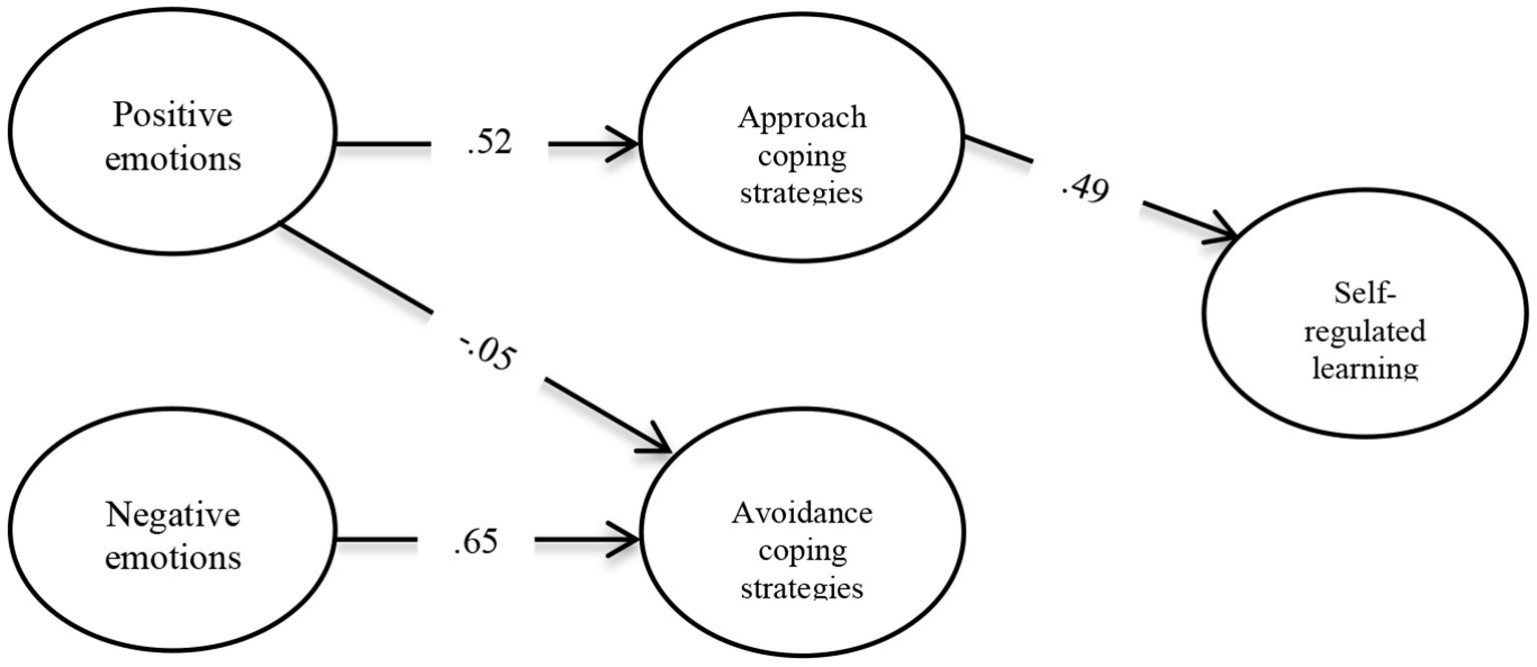
Estimated mediation model of coping strategies between emotions and self-regulation of learning. All parameters were statistically significant at *p* < 0.001, except the effect of positive emotions and avoidance coping strategies, which was significant at *p* < 0.05.

Figure 2 summarizes the significant results. Data indicate that not all hypotheses were fully supported. Specifically, the first hypothesis (H1) was confirmed; students' positive emotions are positively correlated with approach coping strategies (β = 0.52, *t* = 19.87) and negatively with avoidance coping strategies (β = −0.05, *t* = 2.26). The second hypothesis (H2) was partially confirmed; students' negative emotions are positively correlated with avoidance coping strategies (β = 0.65, *t* = 37.40) but not with approach coping strategies. The third hypothesis (H3) was partially confirmed; students' self-regulated learning is positively correlated with the approach coping strategies (β = 0.49, *t* = 22.03) but not with the avoidance coping strategies. Finally, the fourth hypothesis (H4) was partially confirmed; students' approach coping strategies partially mediate the relationship between positive emotions and self-regulated learning (β = 0.25, *t* = 14.13). However, there is not a significant relationship between avoidance coping strategies and self-regulated learning.

## Discussion

This study aimed to analyze the relationships between students' emotions, coping strategies, and self-regulated learning in online learning during COVID-19 home confinement. This section discusses the main findings of the study.

### Students' Living Conditions and Emotional State During Confinement

Results showed that most participants lived in urban areas during COVID-19 home confinement and shared living quarters with their families. Likewise, the majority of students had their own studying space to continue their studies online; with ventilation and natural light, Internet service, computers, and other electronic devices. These findings suggest favorable conditions regarding social support, living conditions, and resources to continue studying online.

As expected in all pandemics (Taylor, 2019), anxiety and frustration were present among participants during home confinement, however, the primary emotions were gratitude, joy, and hope. Emotions such as fear, loneliness, and lack of interest were less present among participants. These results suggest that, although pandemic uncertainty and the lockdown conditions, as well as the academic demands, generated high levels of stress and anxiety in university students (Orellana and Orellana, 2020; González-Jaimes et al., 2021), this situation also favored positive personal experiences (Johnson et al., 2020; Sandin et al., 2020). Contrary to other research on this population (Johnson et al., 2020), fear had not been one of the university students' most frequent emotions.

In this sense, it is important to consider that, to a certain degree, anxiety helps one cope with situations and modify behavioral patterns to cope with uncertain situations, but in a more significant way, it may also be a risk to health and well-being (WHO, 2020). Participants' positive emotion perceptions, such as hope and joy, and relational emotions, such as gratitude, seem to be associated with the social resources and materials that most of the students had during the lockdown.

As indicated by previous research, living with parents in urban areas, with proper living conditions (Cao et al., 2020) are protective factors for students faced with anxiety caused by uncertainty and social distancing, which would seem to apply to the context of this study. According to Tipismana (2019), together with some personal factors, family support is a significant predictor of academic variables such as achievement expectations, perceived learning, and global satisfaction. Moreover, having an Internet connection (Baloran, 2020) and the commodities and devices necessary to study online contribute to most university students' stress and anxiety mitigation.

### Coping Strategies Used by University Students

Regarding the main coping strategies used by participants, results showed that 74% of students used positive reappraisal of the situation through looking for the most favorable aspects of confinement, in terms of realizing what was essential and seeing that there were good things, as well as people who worried about them. Second, they reported having put cognitive coping approach strategies into practice by saying encouraging messages, thinking they are better off than others in the same situation and that things could be worse. These findings coincide with previous research (Piergiovanni and Depaula, 2018) in which, in light of situations of stress and feeling loss of control, the majority of the students tried to face the situation by focusing on positive aspects.

Likewise, most of the students mentioned using focalization on problem-solving strategies (64%), analyzing the causes of the situation to face it, and making an action plan. These findings coincide with those of Vidal-Conti et al. (2018) and De La Rosa-Rojas et al. (2015), in which the actions carried out by university students to restore the systemic balance caused by academic stressors focused on solving the troublesome situation, by creating an action plan for concentrating on their homework.

A similar percentage of students using cognitive coping approach strategies (67%) indicated having used avoidance strategies to not think about the problem, such as watching movies or series and going for a walk or run. Another way of coping with the situation by several students (63%) was behavioral avoidance strategies such as reading, listening to music, and finding other ways to enjoy life. In general, these findings coincide with those found by Baloran (2020) upon assessing behavioral-type strategies in students, observing that participation in relaxing activities, such as sports, exercise, and music during the pandemic lockdown were beneficial.

The main coping strategies used by students were active, focused on positive reappraisal, contrasting styles more oriented to seeking social support, spirituality, and negative self-focalization. However, most students also reported behavioral avoidance strategies in the face of social distancing. Considering the frequency with which each type of strategy was used, the findings presented here are very similar to those reported by Piergiovanni and Depaula (2018), in that, after positive reappraisal, negative self-focalization, open emotional expression, avoidance, seeking social support, and, to a lesser degree, turning to religion are frequently seen in university students.

The fact that participants used more strategies focused on the problem than on emotions may reflect that, even though the pandemic as a situation is not within their control, students considered themselves capable of responding to the challenges of the situation, although, their family and living conditions may have helped. In this sense, for several decades Billings and Moos (1981) have emphasized the role of social resources in the coping strategies that are put into practice, which may complement personal resources. However, in threatening situations, people tend to present a combination of several emotional reactions (Lazarus and Folkman, 1984). In this study, religion functioned more as an emotional resource, while in the Spanish context it constituted more of a behavioral coping strategy that enabled people to focus on the problem (González and Landero, 2007).

### Relationship Between Emotions, Coping Strategies, and Self-Regulated Learning

Results show that cognitive coping approach strategies, which imply thinking favorably about the situation and its consequences, maintain a positive relationship with emotions such as compassion, hope, gratitude, and joy. The former was also associated with emotions such as fear and confusion, although with less strength. These findings indicate that thinking positively about the pandemic bolsters positive emotions, but being conscious of its uncertainty may also generate negative emotions. It should be mentioned that these strategies were positively related to positive reappraisal strategies, having in common the possibility to make sense of the situation. As Li and Peng* (2020) study indicates, cognitive coping, like emotional coping and social support, helps decrease anxiety.

Cognitive avoidance strategies, which involve thoughts of not focusing directly on the problem, are positively associated with negative emotions and negatively to tranquility. Chao (2011) indicates that the use of strategies focused on avoidance was related to a lesser degree to psychological well-being and the presence of greater negative affectivity. Simultaneously, behavioral avoidance coping strategies, which would imply carrying out activities to take one's mind off the situation, maintained a positive relationship both with positive and negative emotions, although with little strength.

More precisely, it was a positive relationship between focalization on problem-solving and positive reappraisal strategies with all the positive emotions (compassion, tranquility, hope, gratitude, and joy). There was also a positive relationship between negative self-focalization and open emotional expression strategies and all the negative emotions (boredom, loneliness, anger, frustration, fear, confusion, sadness, anxiety/concern, and despair). It should be mentioned that these two strategies, which are more broadly seen as emotional strategies (Sandín and Chorot, 2003), imply that things will go wrong, and being irritable with others, showed a negative relationship with tranquility.

Regarding behavioral coping approach strategies, which imply asking for help, a positive relationship with emotions such as confusion, fear, and despair was found, and to a lesser degree, there was a relationship with anxiety/concern, loneliness, compassion, and frustration. This result implies that, even when dealing with negative emotions, there is a distinction in its expression and management, supporting Lazarus and Folkman's coping theory (1984).

At the same time, seeking social support was positively related to the emotions associated with uncertainty, such as fear and confusion, compassion, sadness, despair, and, to a lesser degree, to some more favorable emotions such as gratitude and joy. These findings suggest that it is less probable that students seek help from friends and family when they feel angry, frustrated, bored, or alone, but they may seek help if experiencing fear or confusion during the lockdown. In this regard, the results of Li et al. (2020) confirmed that social support has a significant negative predictive effect on anxiety, which is also present during pandemics. Finally, turning to religion as a strategy to cope with lockdown was positively related to hope, joy, gratitude, compassion, and fear.

Results also showed that the coping strategies used through a cognitive approach, cognitive avoidance, and behavioral avoidance correlated significantly with all self-regulated learning dimensions, the first and third positively, and the second negatively; in the first case, by focusing on the situation and generating positive thoughts about it, whereas in the second case, thoughts have a negative component. In the third case, the person involves him or herself in activities that contribute to more pleasant experiences of living in adverse situations.

Focalization on problem-solving and positive reappraisal were the coping strategies with the strongest association to each of the self-regulated learning dimensions. Both are considered generic strategies, focused on rationality and showing a positive relationship with cognitive coping approach strategies. While positive reappraisal focuses on using concrete actions to correctly manage the situation, a positive sense of what is being lived is given in positive reappraisal. De La Rosa-Rojas et al. (2015) stated that problem-solving strategies or the search for information are active coping behaviors and are most frequently associated with greater competence and positive functioning.

A proactive approach to seeking social support was related to all the self-regulated learning dimensions, although the relationship's strength was meager. This type of strategy, as shown in the literature (Karabenick and Gonida, 2018), is based on searching for support from classmates, teachers or other professionals, and even family members, and has a lot in common with other self-regulated learning strategies, when facing a lack of progress toward the achievement of the desired academic goals. It should be noticed that, although turning to religion correlated significantly with the different dimensions of self-regulated learning, the strength of the relationship was also low. Finally, as was to be expected, negative self-focalization and open emotional expression, which imply poor emotional management, showed a negative relationship with all the dimensions of self-regulated learning. This same inverse relationship happened in cognitive avoidance strategies, which may imply negation or an unrealistic view of the situation.

### Mediation of Coping Strategies Between Emotions and Self-Regulation of Learning

This study focused on the role of coping as a mediating agent in the relationship between emotions and student self-regulated learning. In general, results show the mediating role of approach coping strategies between emotions and self-regulated learning, but there is not a significant relationship between avoidance coping strategies and self-regulated learning. According to the theoretical bases on coping on which this study is based (Billings and Moos, 1981; Lazarus and Folkman, 1986; Lazarus, 2006), active strategies, such as problem-centered strategies, tend to be most desirable (Folkman and Lazarus, 1980), while avoidance strategies are the least desirable (Lazarus, 2006). Based on the results of this study, it is important to underline the positive impact active strategies have on self-regulated learning processes (planning, monitoring, control, and reflection) that allow students to get involved in and achieve learning in online environments.

The fact that positive emotions are associated with self-regulated learning, while negative emotions are not, is consistent with previous research (De la Fuente et al., 2020), indicating that maintaining positive emotional states promotes more significant learning involvement. Likewise, as Hendrie and Bastacini (2020) state, positive and negative emotions influence people's judgment of their performance. It is, therefore, necessary to consider aspects of a student's cognitive and emotional ability in relation to their academic involvement.

Regarding the role of participants as students and given that self-regulated learning indicators are observed, we can infer that students recognized themselves as capable of handling situations such as fulfilling assignments, and connecting to synchronous sessions, etc. This is complemented by the possibility of seeking support from others, as an additional resource for their management, also favored by the technological resources available. Moreover, these findings suggest that, in general, participants had personal resources, allowing them to reduce their vulnerability to threats and favor more effective coping.

These findings are novel because they offer clear evidence about the positive association between self-regulated learning and approach coping strategies (De la Fuente et al., 2015). On the contrary, avoidance coping strategies, which imply not thinking about the problem and poor emotional management, do not contribute to committing oneself and making an effort to learn while faced with circumstances involved in the pandemic lockdown. Therefore, we can conclude that approach coping strategies focused on the problem and personal reappraisal promote self-regulated learning, while avoidance coping strategies, such as not thinking about worries, inhibit it.

In summary, this research has shown, together with the findings by Lazarus and Lazarus (2000), that approach (active) coping strategies guided toward a positive reappraisal of the situation (CCA, PRE, CAC), contribute to personal well-being, in this case, to mitigate the effects of lockdown during the pandemic. At the same time, the focalization on problem-solving (FPS) coping strategy and analyzing the causes of the situation in order to face it, allow the students to carry out different strategies, ones they have tried in the past, or to implement new ones, to counteract adverse effects and continue with their studies online.

If lockdown has created anxiety, frustration, and boredom in students, emotions such as tranquility, hope, gratitude, and joy are the result of a positive reappraisal of the importance initially given to the pandemic, as well as performing different activities that bring out the most favorable aspects of the situation, and maintain performance during online academic activities. Therefore, activating these coping strategies constitutes a key mechanism to positively face problems and challenges, generating positive emotions essential for the self-regulation of learning. Simultaneously, such strategies help protect them from emotions such as loneliness and lack of interest, impeding academic involvement in a self-regulated manner. In light of the results presented, we consider that this study contributes to the corpus of knowledge on the study of self-regulated learning as a complex process in which the affective, motivational, cognitive, and behavioral aspects are closely related (Zimmerman, 2008; De la Fuente et al., 2015). Therefore, approach coping strategies via the mediating role they play in generating positive emotions are fundamental to this process.

These results allow for the identification of important educational implications. From our findings, we consider it essential that professors help university students understand the relevance of active coping strategies for the better management of uncertain circumstances out of their control, which they are currently experiencing due to the pandemic. It is also necessary to consider each student's reality and the use of virtual teaching and learning models focused on the student that promote autonomous and self-regulated learning in environments of warmth and acknowledgment of the other. Today more than ever, universities and teachers should be creative in supporting the students' educational processes properly, using their resources to respond to the students' affective and learning needs from the new challenges the pandemic has left.

Despite the good results, one aspect to highlight is that the majority of the students who took part in this study lived with their families in urban areas and with relatively adequate living conditions to continue their studies online during lockdown; therefore, it is inferred that these aspects constitute one more factor in mitigating students' anxiety caused by uncertainty and social distancing. We believe it necessary in future research to increase the number of participants from different regions of the country, especially students from semi-urban and rural areas. The results also pose new questions regarding the possible use of approach coping strategies and self-regulated learning processes based on the students' profile. Another essential aspect of future research would involve a comparison among universities in different countries to enable a transcultural analysis of the variables seen in this study, which would enable a greater understanding of the needs of university students in a situation of this magnitude, allowing teaching staff to support their emotional well-being and the achievement of their academic goals during and after lockdown.

## Data Availability Statement

The raw data supporting the conclusions of this article will be made available by the authors, without undue reservation.

## Ethics Statement

The studies involving human participants were reviewed and approved by Universidad Popular Autónoma del Estado de Puebla Ethics Committee. Written informed consent for participation was not required for this study in accordance with the national legislation and the institutional requirements.

## Author Contributions

MLG analyzed the data and drafted the initial version of the article. All authors contributed to the conception and design of the study, data collection, results interpretation, and approval of the final version.

## Conflict of Interest

The authors declare that the research was conducted in the absence of any commercial or financial relationships that could be construed as a potential conflict of interest.
